# Function of RSKS-1-AAK-2-DAF-16 signaling cascade in enhancing toxicity of multi-walled carbon nanotubes can be suppressed by *mir-259* activation in *Caenorhabditis elegans*

**DOI:** 10.1038/srep32409

**Published:** 2016-08-30

**Authors:** Ziheng Zhuang, Min Li, Hui Liu, Libo Luo, Weidong Gu, Qiuli Wu, Dayong Wang

**Affiliations:** 1 School of Pharmaceutical Engineering and Life Sciences, Changzhou University, Changzhou 213164, China; 2 Changzhou No. 7 People’s Hospital, Changzhou 213011, China; 3 Key Laboratory of Environmental Medicine Engineering in Ministry of Education, Medical School, Southeast University, Nanjing 210009, China

## Abstract

*Caenorhabditis elegans* is an important non-mammalian alternative assay model for toxicological study. Previous study has indicated that exposure to multi-walled carbon nanotubes (MWCNTs) dysregulated the transcriptional expression of *mir-259*. In this study, we examined the molecular basis for *mir-259* in regulating MWCNTs toxicity in nematodes. Mutation of *mir-259* induced a susceptible property to MWCNTs toxicity, and MWCNTs exposure induced a significant increase in *mir-259::GFP* in pharyngeal/intestinal valve and reproductive tract, implying that *mir-259* might mediate a protection mechanisms for nematodes against MWCNTs toxicity. RSKS-1, a putative ribosomal protein S6 kinase, acted as the target for *mir-259* in regulating MWCNTs toxicity, and mutation of *rsks-1* suppressed the susceptible property of *mir-259* mutant to MWCNTs toxicity. Moreover, *mir-259* functioned in pharynx-intestinal valve and RSKS-1 functioned in pharynx to regulate MWCNTs toxicity. Furthermore, RSKS-1 regulated MWCNTs toxicity by suppressing the function of AAK-2-DAF-16 signaling cascade. Our results will strengthen our understanding the microRNAs mediated protection mechanisms for animals against the toxicity from certain nanomaterials.

Multi-walled carbon nanotubes (MWCNTs), one member of the carbon nanotubes (CNTs), have numerous unique physicochemical properties. So far, MWCNTs have been produced in bulk for diverse purposes. With the increase in MWCNTs manufacture, it is likely that the increasing exposure of human and environmental organisms to MWCNTs will occur^1^,^2^. The potential toxic effects of ENMs including the MWCNTs on organisms have received the great attentions^3^,^4^,^5^. It has been shown that exposure to CNTs can lead to several aspects of toxicity on organisms through the induction of oxidative stress and/or inflammation and crossing the biological barriers^6^,^7^,^8^,^9^. To determine the underlying molecular mechanisms of MWCNTs toxicity, some dysregulated genes or microRNAs (miRNAs) have been identified in mice or NIH/2Ts cells^10^,^11^. miRNAs, a class of short noncoding RNAs, usually exhibit their biological functions by post-transcriptionally inhibiting the expression of targeted genes^12^.

Due to the properties of short lifespan, ease of manipulation, and especially the well-described genetic background^13^,^14^,^15^, the important non-mammalian alternative toxicity assay model of *Caenorhabditis elegans* has been recently used in the toxicological study of MWCNTs. In nematodes, MWCNTs exposure could cause the damage on the functions of both primary targeted organs such as intestine and secondary targeted organs such as neurons and reproductive organs^16^,^17^,^18^,^19^. MWCNTs could be further translocated into the secondary targeted organs such as the reproductive organs in nematodes^16^,^17^,^20^. Moreover, based on the SOLiD sequencing, some dysregulated miRNAs have been identified in MWCNTs exposed nematodes^21^. Biological functions of some dysregulated miRNAs in regulating MWCNTs toxicity have been confirmed with the aid of the available mutants^21^. Nevertheless, the molecular signaling pathways mediated by these candidate miRNAs in regulating MWCNTs toxicity are still largely unclear in nematodes.

Previous study has demonstrated that MWCNTs exposure increased the expression of *mir-259* in nematodes^21^. In nematodes, mutation of *mir-259* induced a susceptible property to MWCNTs toxicity^21^. The MWCNTs (1 mg/L) exposed *mir-259*(*n4106)* mutant exhibited a more significant reduction in brood size, decrease in locomotion behavior, and induction of intestinal autofluorescence or reactive oxygen species (ROS) production than MWCMTs exposed wild-type nematodes^21^. However, the underlying epigenetic mechanism for *mir-259* in regulating MWCNTs toxicity is still unclear. In this study, we investigated the molecular signaling mediated by *mir-259* in regulating MWCNTs toxicity in the *in vivo* assay system of *C. elegans*. In nematodes, *rsks-1* gene encodes a putative ribosomal protein S6 kinase (S6K) that is required for the longevity control^22^, *aak-2* gene encodes a catalytic alpha subunit of AMP-activated protein kinases (AMPKs), and *daf-16* gene encodes a FOXO transcriptional factor in the insulin signaling pathway. We raised a RSKS-1-AAK-2-DAF-16 signaling cascade mediated by *mir-259* in the control of MWCNTs toxicity in nematodes. Our study reveals the key function of *mir-259* mediated signaling cascade in encoding a protection mechanism for nematodes against the MWCNTs toxicity.

## Results

### Effects of MWCNTs exposure on lifespan and locomotion behavior during aging in *mir-259* mutant nematodes

**Figure 1:**
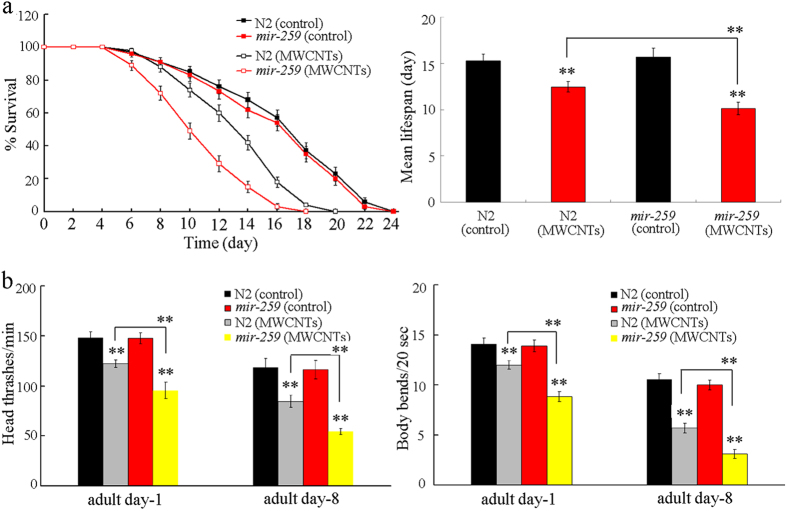
Effects of MWCNTs exposure on lifespan and locomotion behavior during aging in nematodes. (**a**) Effects of MWCNTs exposure on lifespan in nematodes. (**b**) Effects of MWCNTs exposure on locomotion behavior during aging in nematodes. Exposure concentration of MWCNTs was 1 mg/L. Prolonged exposure was performed from L1-larvae to young adults. Bars represent means ± SD. ^**^P < 0.01 *vs* control (if not specially indicated).

Lifespan can reflect the long-term effect of certain toxicants on animals. *mir-259*(*n4106)* has the similar lifespan to that in wild-type nematodes (Fig. 1a). After prolonged exposure, MWCNTs (1 mg/L) exposed *mir-259*(*n4106)* mutant exhibited the significantly reduced lifespan than MWCNTs (1 mg/L) exposed wild-type nematodes (Fig. 1a).

Moreover, we selected the endpoint of locomotion behavior to assess the change of aging-related property during aging in MWCNTs exposed nematodes^23^. *mir-259*(*n4106)* has the similar head thrash and body bend to those in wild-type nematodes (Fig. 1b). After prolonged exposure, MWCNTs (1 mg/L) exposed *mir-259*(*n4106)* mutant showed the significantly decreased head thrash or body bend at adult day-8 than MWCNTs (1 mg/L) exposed wild-type nematodes (Fig. 1b). Therefore, MWCNTs exposure may cause the adverse effects on lifespan and aging related properties such as locomotion behavior during aging in nematodes.

### Effect of MWCNTs exposure on spatial expression of *mir-259* in nematodes

**Figure 2:**
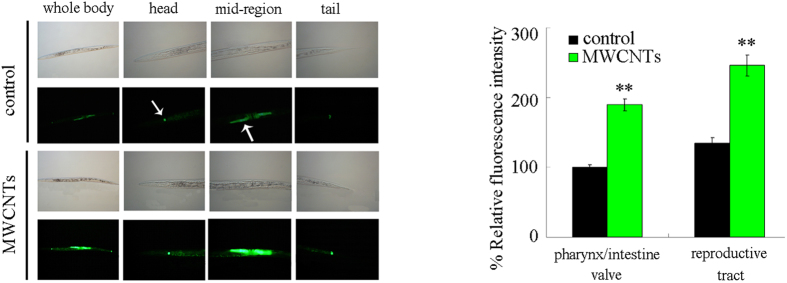
Effects of MWCNTs exposure on *mir-259::GFP* expression in nematodes. Arrowheads indicate pharyngeal/intestinal valve in the head and reproductive tract in the mid-region, respectively. Exposure concentration of MWCNTs was 1 mg/L. Prolonged exposure was performed from L1-larvae to young adults. Bars represent means ± SD. ^**^P < 0.01 *vs* control.

With the aid of transgenic strain of *maIs268*[*mir-259::GFP*], we investigated the effect of MWCNTs exposure on spatial expression of *mir-259::GFP*. In nematodes, *mir-259* is expressed in pharyngeal/intestinal valve and reproductive tract^24^. After prolonged exposure, MWCNTs (1 mg/L) significantly increased the fluorescence intensity of *mir-259::GFP* in pharyngeal/intestinal valve and reproductive tract compared in nematodes (Fig. 2).

### Tissue-specific activity of *mir-259* in regulating MWCNTs toxicity in nematodes

**Figure 3:**
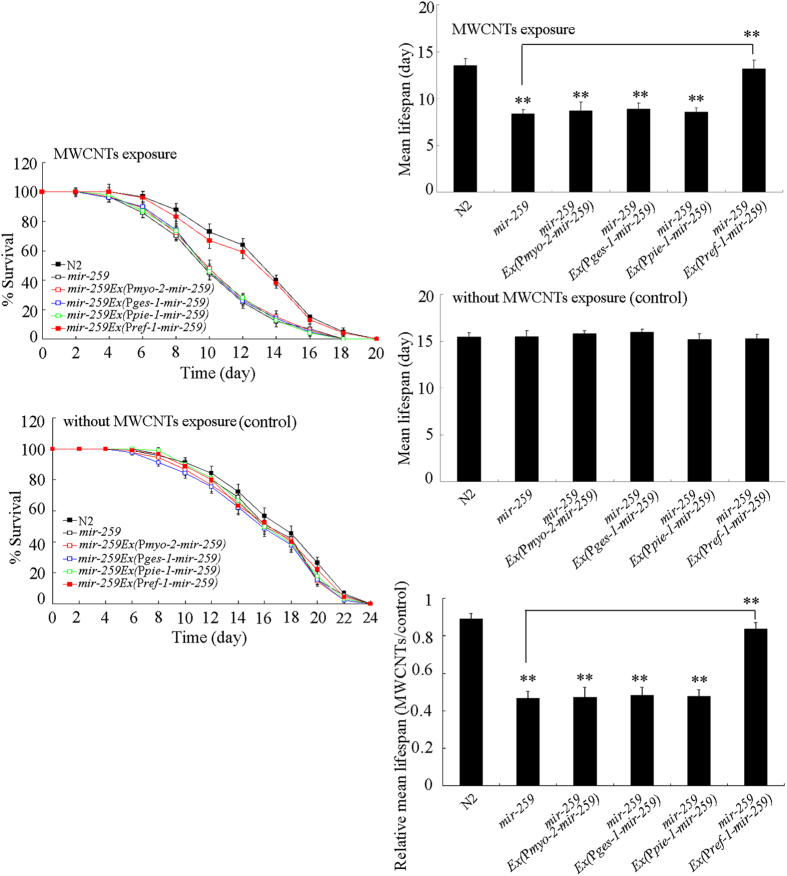
Tissue-specific activity of *mir-259* in regulating MWCNTs toxicity on lifespan in nematodes. Exposure concentration of MWCNTs was 1 mg/L. Prolonged exposure was performed from L1-larvae to young adults. Bars represent means ± SD. ^**^P < 0.01 *vs* N2 (if not specially indicated).

With the aid of tissue-specific promoters, we next investigated the tissue-specific activity of *mir-259* in regulating MWCNTs toxicity in nematodes. Rescue assay by expression of *mir-259* in intestine, pharynx, or reproductive tract did not significantly influence the susceptible property of *mir-259*(*n4106)* mutant nematodes to MWCNTs toxicity on lifespan (Fig. 3). In contrast, expression of *mir-259* in pharynx/intestinal valve could significantly suppress the susceptible property of *mir-259*(*n4106)* mutant nematodes to MWCNTs toxicity on lifespan (Fig. 3). These results imply that *mir-259* may act in the pharynx/intestinal valve to regulate MWCNTs toxicity in nematodes.

### *rsks-1* might act as the potential targeted gene for *mir-259* in nematodes

**Figure 4:**
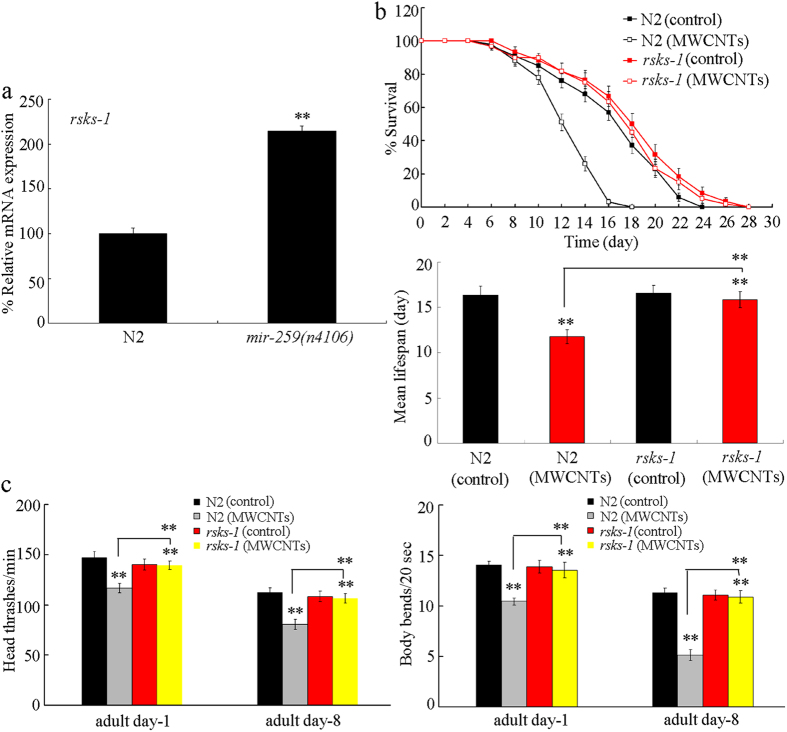
Effects of *rsks-1* mutation on MWCNTs toxicity in nematodes. (**a**) Effect of *mir-259* mutation on expression of *rsks-1* gene. Bars represent means ± SD. ^**^P < 0.01 *vs* N2. (**b**) Effects of *rsks-1* mutation on MWCNTs toxicity in reducing lifespan in nematodes. Bars represent means ± SD. ^**^P < 0.01 *vs* control (if not specially indicated). (**c**) Effects of *rsks-1* mutation on MWCNTs toxicity in decreasing locomotion behavior in nematodes. Bars represent means ± SD. ^**^P < 0.01 *vs* control (if not specially indicated). Exposure concentration of MWCNTs was 1 mg/L. Prolonged exposure was performed from L1-larvae to young adults.

Using the TargetScan tool, we found that *mir-259* may function as an upstream regulator for *rsks-1* gene by binding its 3′-UTR. In the loss-of-function *mir-259*(*n4106)* mutant, the expression of *rsks-1* gene was significantly increased compared with that in wild-type nematodes (Fig. 4a), implying that *mir-259* may inhibit the expression of *rsks-1* gene.

### RSKS-1 was involved in the control of MWCNTs toxicity in nematodes

Using the loss-of-function *rsks-1*(*ok1255)* mutant, we investigated the potential function of RSKS-1 in the control of MWCNTs toxicity in nematodes. The *rsks-1*(*ok1255)* mutant has the similar lifespan and locomotion behavior to those in wild-type nematodes (Fig. 4b,c). After prolonged exposure, the MWCNTs (1 mg/L) exposed *rsks-1*(*ok1255)* mutant exhibited the similar lifespan and locomotion behavior at adult day-8 to those in *rsks-1*(*ok1255)* mutant or wild-type nematodes without MWCNTs exposure (Fig. 4b,c). That is, the *rsks-1*(*ok1255)* mutant had the resistant property to MWCNTs toxicity in nematodes.

### Genetic interaction between *mir-259* and *rsks-1* in regulating MWCNTs toxicity in nematodes

**Figure 5:**
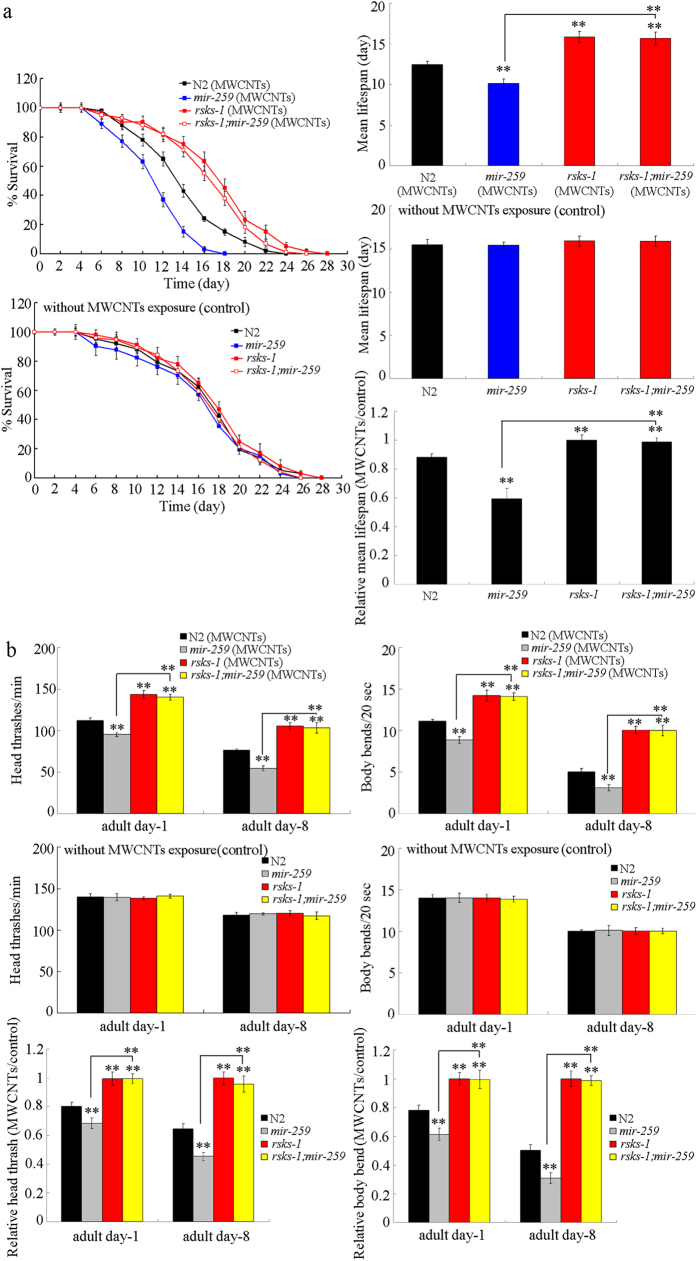
Genetic interaction between *mir-259* and *rsks-1* in regulating MWCNTs toxicity in nematodes. (**a**) Genetic interaction between *mir-259* and *rsks-1* in regulating MWCNTs toxicity in reducing lifespan in nematodes. (**b**) Genetic interaction between *mir-259* and *rsks-1* in regulating MWCNTs toxicity in decreasing locomotion behavior in nematodes. Exposure concentration of MWCNTs was 1 mg/L. Prolonged exposure was performed from L1-larvae to young adults. Bars represent means ± SD. ^**^P < 0.01 *vs* N2 (if not specially indicated).

To confirm the interaction between *mir-259* and *rsks-1* in regulating MWCNTs toxicity, we compared the MWCNTs toxicity in double mutant of *rsks-1*(*ok1255);mir-259*(*n4106)* with that in single mutant of *mir-259*(*n4106)* or *rsks-1*(*ok1255)*. After MWCNTs (1 mg/L) exposure, the lifespan and locomotion behavior at adult day-8 in double mutant of *rsks-1*(*ok1255);mir-259*(*n4106)* were similar to those in single mutant of *rsks-1*(*ok1255)* (Fig. 5), implying that the susceptible property of *mir-259*(*n4106)* mutant to MWCNTs toxicity on lifespan and aging-related properties could be suppressed by *rsks-1* mutation in nematodes. Therefore, *mir-259* may inhibit the function of RSKS-1 in positively regulating the MWCNTs toxicity in nematodes.

### Tissue-specific activity of *rsks-1* in regulating MWCNTs toxicity in nematodes

**Figure 6:**
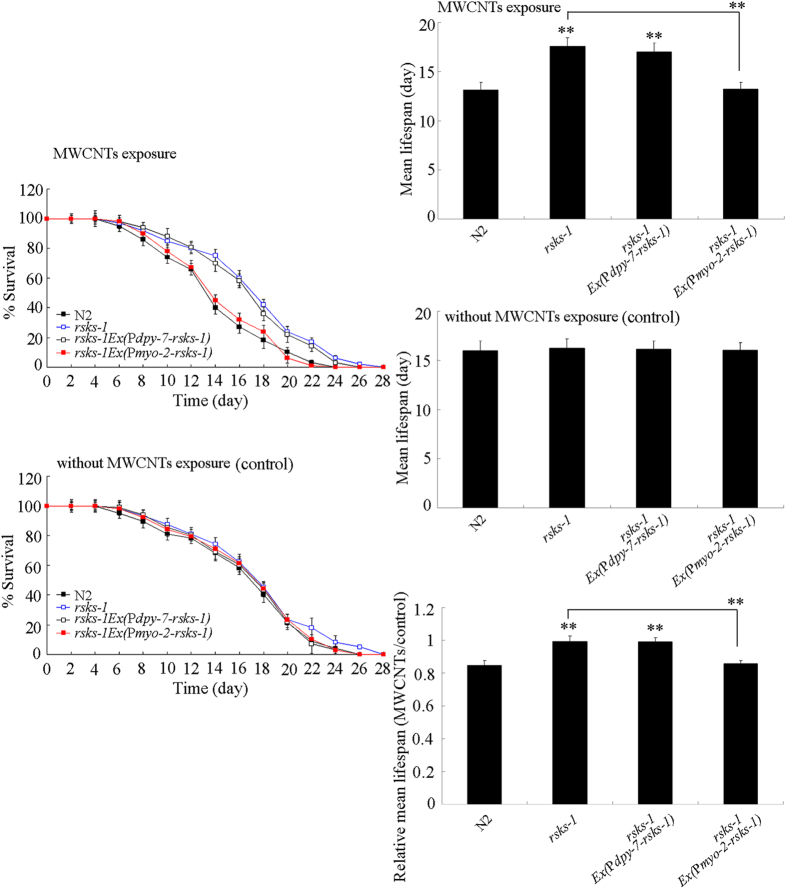
Tissue-specific activity of *rsks-1* in regulating MWCNTs toxicity on lifespan in nematodes. Exposure concentration of MWCNTs was 1 mg/L. Prolonged exposure was performed from L1-larvae to young adults. Bars represent means ± SD. ^**^P < 0.01 *vs* N2 (if not specially indicated).

In *C. elegans*, *rsks-1* gene is expressed in pharynx and hypodermis^16^. Using the tissue-specific promoters, we investigated the tissue-specific activity of *rsks-1* in regulating MWCNTs toxicity in nematodes. Rescue assay by expression of *rsks-1* in hypodermis did not significantly influence the lifespan in MWCNTs (1 mg/L) exposed *rsks-1*(*ok1255)* mutant nematodes (Fig. 6). In contrast, expression of *rsks-1* in pharynx could significantly decrease the lifespan in MWCNTs (1 mg/L) exposed *rsks-1*(*ok1255)* mutant nematodes (Fig. 6). These results suggest that RSKS-1 may act in the pharynx to regulate MWCNTs toxicity in nematodes.

### Genetic interaction between *rsks-1* and *aak-2* in regulating MWCNTs toxicity in nematodes

**Figure 7:**
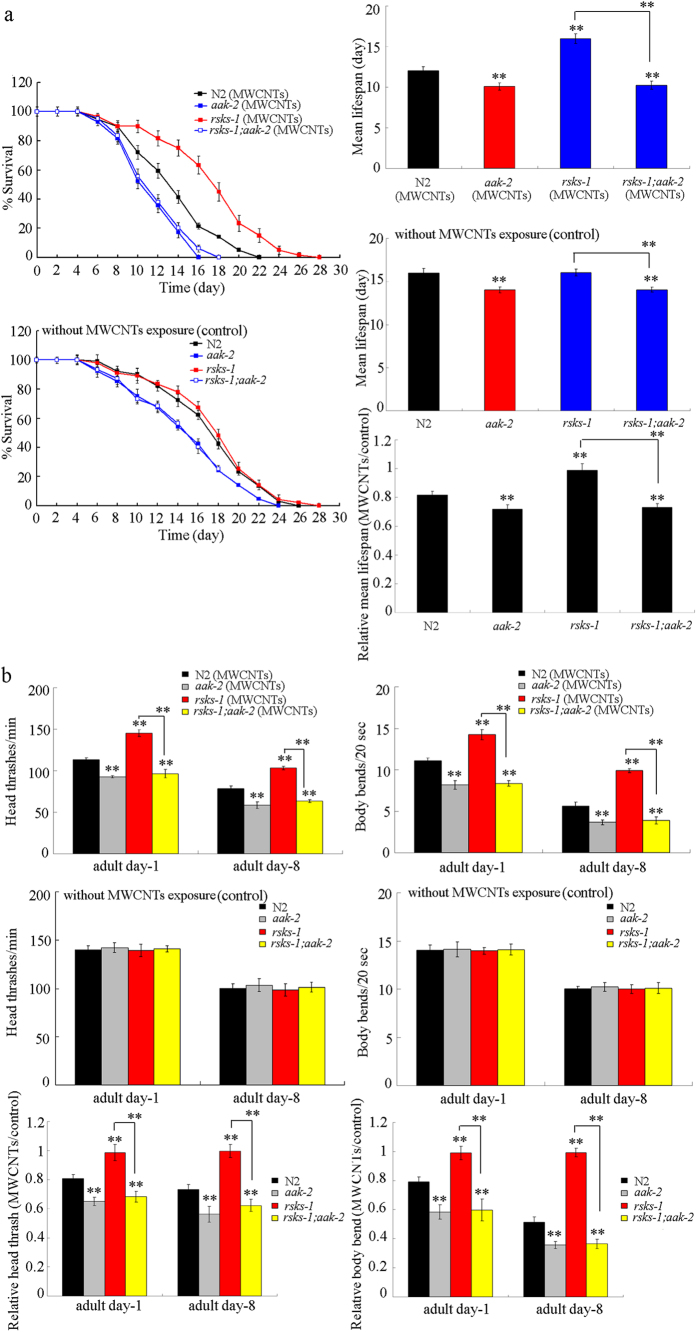
Genetic interaction between *rsks-1* and *aak-2* in regulating MWCNTs toxicity in nematodes. (**a**) Genetic interaction between *rsks-1* and *aak-2* in regulating MWCNTs toxicity in reducing lifespan in nematodes. (**b**) Genetic interaction between *rsks-1* and *aak-2* in regulating MWCNTs toxicity in decreasing locomotion behavior in nematodes. Exposure concentration of MWCNTs was 1 mg/L. Prolonged exposure was performed from L1-larvae to young adults. Bars represent means ± SD. ^**^P < 0.01 *vs* N2 (if not specially indicated).

Previous study has implied that AAK-2 may act as an important molecular target for RSKS-1 in regulating longevity^22^. To determine whether RSKS-1 can act though the AAK-2 mediated signaling to regulate MWCNTs toxicity, we compared the MWCNTs toxicity in double mutant of *rsks-1*(*ok1255);aak-2*(*ok524)* with that in single mutant of *rsks-1*(*ok1255)* or *aak-2*(*ok524)*. After MWCNTs (1 mg/L) exposure, the lifespan and locomotion behavior at adult day-8 in double mutant of *rsks-1*(*ok1255);aak-2*(*ok524)* were similar to those in *aak-2*(*ok524)* mutant nematodes (Fig. 7), suggesting that the resistant property of *rsks-1*(*ok1255)* mutant to MWCNTs toxicity on lifespan and aging-related properties could be inhibited by *aak-2* mutation in nematodes. These results imply that RSKS-1 may genetically act upstream of AAK-2 to regulate MWCNTs toxicity in nematodes.

### Genetic interaction between *aak-2* and *daf-16* in regulating MWCNTs toxicity in nematodes

**Figure 8:**
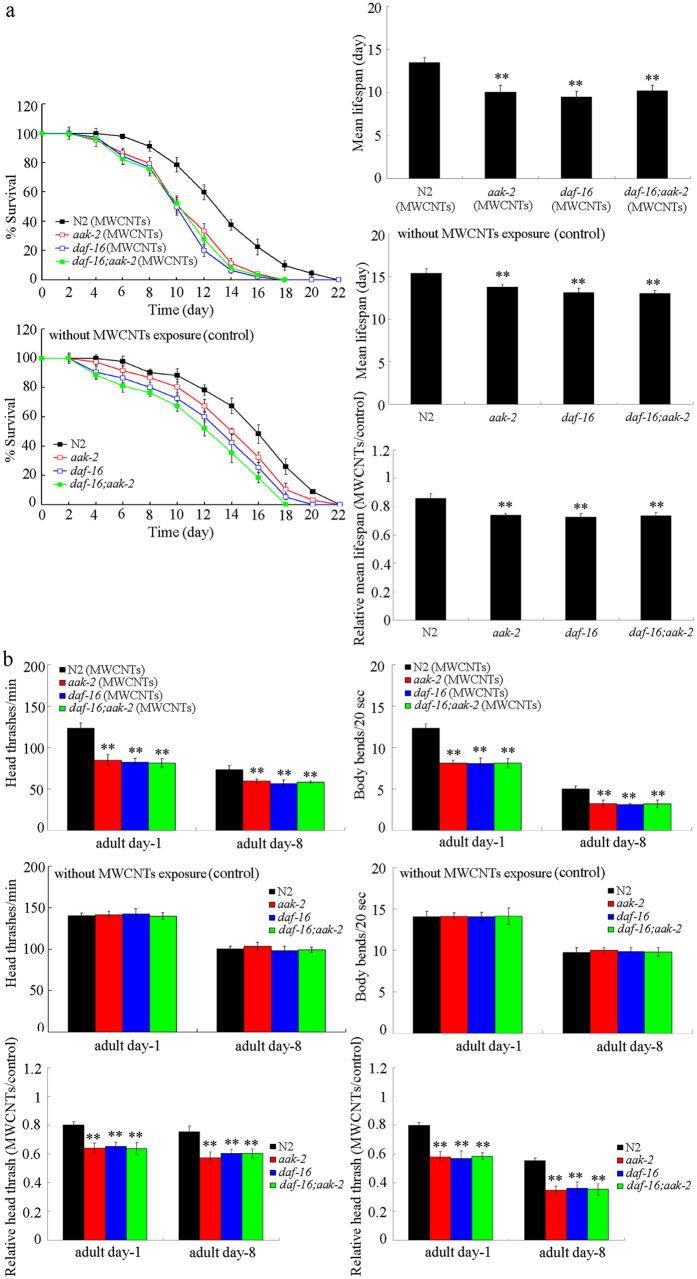
Genetic interaction between *aak-2* and *daf-16* in regulating MWCNTs toxicity in nematodes. (**a**) Genetic interaction between *aak-2* and *daf-16* in regulating MWCNTs toxicity in reducing lifespan in nematodes. (**b**) Genetic interaction between *aak-2* and *daf-16* in regulating MWCNTs toxicity in decreasing locomotion behavior in nematodes. Exposure concentration of MWCNTs was 1 mg/L. Prolonged exposure was performed from L1-larvae to young adults. Bars represent means ± SD. ^**^P < 0.01 *vs* N2.

Previous study has further suggested AAK-2 may function upstream of DAF-16 in insulin signaling pathway to regulate biological processes such as longevity in nematodes^22^. To determine the genetic interaction between *aak-2* and *daf-16* in regulating MWCNTs toxicity, we compared the MWCNTs toxicity in double mutant of *daf-16*(*mu86);aak-2*(*om524)* with that in single mutant of *daf-16*(*mu86)* or *aak-2*(*om524)*. After MWCNTs (1 mg/L) exposure, we found that the lifespan and locomotion behavior at adult day-8 in double mutant of *daf-16*(*mu86);aak-2*(*om524)* were similar to those in single mutant of *aak-2*(*om524)* or *daf-16*(*mu86)* nematodes (Fig. 8), implying that AAK-2 can act together with DAF-16 in the same genetic pathway to regulate the MWCNTs toxicity in nematodes.

### Distribution and translocation of MWCNTs in *mir-259*, *rsks-1*, *aak-2*, and *daf-16* mutant nematodes

**Figure 9:**
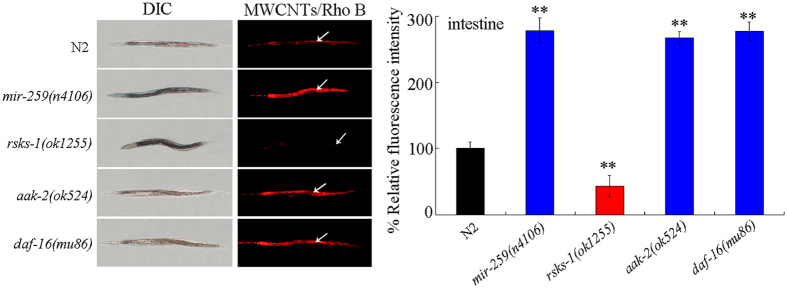
Distribution and translocation of MWCNTs/Rho B in nematodes. Arrowheads indicate the intestine. Exposure concentration of MWCNTs/Rho B was 1 mg/L. Prolonged exposure was performed from L1-larvae to young adults. Bars represent means ± SD. ^**^P < 0.01 *vs* N2.

Biodistribution and translocation are crucial factors for the toxicity formation of ENMs in nematodes^15^. With the aid of molecular probe of Rhodamine B (Rho B), we prepared the MWCNTs/Rho B. After MWCNTs/Rho B exposure, we observed a more pronounced MWCNTs/Rho B distribution in the body of *mir-259*(*n4106), aak-2*(*ok524)*, and *daf-16*(*mu86)* mutants compared with wild-type N2 (Fig. 9). In contrast, mutation of *rsks-1* gene significantly suppressed the distribution of MWCNTs/Rho B in the body of nematodes compared with wild-type N2 (Fig. 9). Exposure to Rho B caused the relatively equal distribution of fluorescence in the tissues of wild-type N2, *mir-259*(*n4106)*, *rsks-1*(*ok1255)*, *aak-2*(*ok524)*, or *daf-16*(*mu86)* mutant nematodes (Fig. S1). Therefore, mutation of the *mir-259*, *aak-2*, or *daf-16* enhanced the biodistribution and translocation of MWCNTs in the body, whereas mutation of *rsks-1* suppressed the accumulation of MWCNTs in the body of nematodes.

## Discussion

Our previous study has demonstrated that, after MWCNTs exposure, loss-of-function mutation of *mir-259* could cause the more significant reduction in brood size, decrease in locomotion behavior, and induction of intestinal autofluorescence or ROS production compared with wild-type nematodes^21^. In this study, after MWCNTs exposure, we further observed that mutation of *mir-259* could result in the more reduced lifespan, and more severely decreased locomotion behavior during the aging (Fig. 1). Therefore, our results suggest that, besides the functions of primary and secondary targeted organs, MWCNTs exposure may further potentially adversely affect the longevity and aging related phenotypes in organisms.

Using the transgenic strain of *maIs268*, we observed that MWCNTs could significantly increase the expression of *mir-259::GFP* in both the pharyngeal/intestinal valve and the reproductive tract (Fig. 2). Meanwhile, we found that the loss-of-function *mir-259* mutant was susceptible to MWCNTs toxicity in nematodes^21^ (Fig. 1). These data imply that *mir-259* may mediate a protection mechanism for nematodes against the MWCNTs toxicity. That is, MWCNTs may induce a protection mechanism encoded by the activated *mir-259* in nematodes. Similarly, previous study has demonstrated that the activated *mir-360* may encode a protection mechanism for nematodes against the toxic effects of graphene oxide (GO) in inducing germline apoptosis^25^.

Previous studies have suggested that some important signaling pathways can act in certain tissues to regulate the toxicity of ENMs in nematodes. For example, *acs-22* gene encoding a protein homologous to mammalian fatty acid transport protein 4 could act in the intestine to regulate MWCNTs toxicity^20^, and insulin signaling pathways could act in the intestine to regulate the GO toxicity^26^. *unc-30* gene encoding a homeodomain transcription factor could act in the RMEs or D-type GABAergic motor neurons to regulate the neurotoxicity of quantum dots (QDs)^27^,^28^. In this study, using the tissue-specific promoters, we found that the *mir-259* acted in the pharynx/intestinal valve to regulate MWCNTs toxicity in nematodes (Fig. 3), implying the potential important function of *mir-259* in affecting the function of pharynx/intestinal valve. We observed that mutation of *mir-259* enhanced the distribution of MWCNTs/Rho B in the body of nematodes (Fig. 9). Moreover, our results further imply the possible important role of pharynx/intestinal valve in the control of toxicity and translocation of certain ENMs in nematodes.

In this study, we provide several lines of evidence to indicate the potential role of RSKS-1 as the target for *mir-259* in regulating the MWCMTs toxicity in nematodes. We observed that the phenotypes in MWCNTs exposed *rsks-1* mutant were opposite to those in MWCNTs exposed *mir-259* mutant. The *rsks-1* mutant was resistant to the MWCNTs toxicity on longevity and aging related phenotypes (Fig. 4). Moreover, we found that mutation of the *rsks-1* gene could suppress the susceptible property of *mir-259*(*n4106)* mutant to MWCNTs toxicity on lifespan and aging-related properties (Fig. 5). Therefore, although we did not exclude the possible involvement of other targets in the control of MWCNTs toxicity, our results indicated that *mir-259* can negatively regulate the MWCNTs toxicity by inhibiting the function of RSKS-1, one of its important targets.

For the tissue-specific activity, our results suggest that *rsks-1* gene acted in the pharynx to regulate the MWCNTs toxicity on longevity and aging related phenotypes in nematodes (Fig. 6). Based on the distribution pattern of MWCNTs/Rho B, we also found that mutation of *rsks-1* significantly suppressed the deposition of MWCNTs in the body of nematodes (Fig. 9). Considering the fact that RSKS-1 acted in the pharynx to regulate the MWCNTs toxicity, the MWCNTs uptake by feeding might be altered in the *rsks-1* mutant nematodes. In contrast, the altered MWCNTs translocation into the secondary targeted organs through the intestinal barrier might be not directly due to the *rsks-1* mutation in nematodes. That is, our data suggest the important role of *rsks-1* gene in the pharynx in regulating the toxicity and accumulation of MWCNTs. Meanwhile, our data also imply the possible crucial role of pharynx in the control of toxicity and accumulation of certain ENMs in nematodes.

In this study, we identified the downstream signaling cascade for RSKS-1 in regulating the MWCNTs toxicity. In *C. elegans*, *aak-2* can function downstream of stressors and energy level signals to positively regulate the adult lifespan^29^. Here we further showed that AAK-2 functioned downstream of RSKS-1 to regulate the MWCNTs toxicity, since mutation of the *aak-2* gene suppressed the resistant property of *rsks-1*(*ok1255)* mutant to MWCNTs toxicity on the longevity and the aging related phenotypes (Fig. 7). Moreover, our results suggest that AAK-2 may further regulate the MWCNTs toxicity on longevity and aging related phenotypes by acting with the transcriptional factor DAF-16 in the same genetic pathway (Fig. 8). Therefore, RSKS-1 may act upstream of the AAK-2-DAF-16 signaling cascade to regulate the MWCNTs toxicity in nematodes. The AAK-2-DAF-16 signaling cascade was also shown to be involved in the longevity regulation in nematodes^22^, implying that the AAK-2-DAF-16 signaling cascade may be a conserved signaling cascade involved in the control of biological processes in nematodes. Nevertheless, RSKS-1, AAK-2, and DAF-16 may not regulate the MWCNTs toxicity by forming a complex, because RSKS-1 acted in the pharynx to regulate the MWCNTs toxicity and DAF-16 normally acted in the intestine to regulate the nanotoxicity and innate immunity^26^,^30^. In addition, previous studies have only shown that AAK-2 can physically interact with ICD-1, ICD-2, F49E8.7, or UNC-42 to regulate biological processes in nematodes (http://www.wormbase.org/species/c_elegans/gene/WBGene00020142#01-9g8-10).

**Figure 10:**
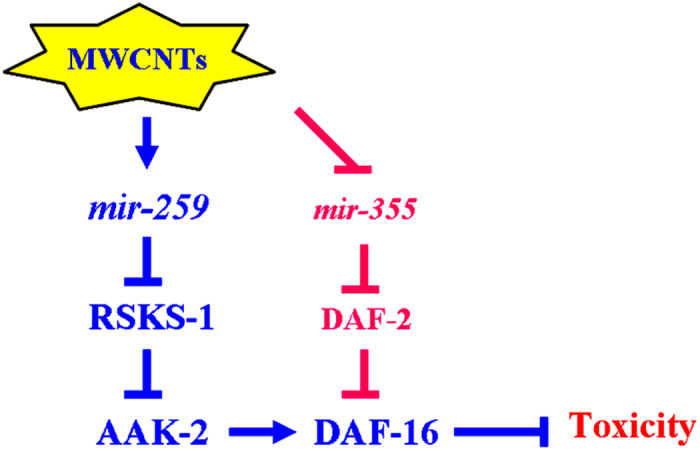
A diagram showing the *mir-259*-mediated molecular signaling in the control of MWCNTs toxicity in nematodes.

In *C. elegans*, the insulin receptor DAF-2 can reduce the longevity or enhance the toxicity of certain toxicants such as MWCNTs by suppressing the expression and function of DAF-16^31^,^32^,^33^. Our recent study has demonstrated that MWCNTs exposure would inhibit the expression of *mir-355*, which further induced the toxic effects of MWCNTs on nematodes by suppressing the function of DAF-2^33^. Therefore, on the one hand, MWCNTs may suppress the function of DAF-16 in inducing the toxicity of MWCNTs on nematodes by inhibiting the expression of *mir-355*; on the other hand, MWCNTs may be also able to potentially enhance the function of DAF-16 in decreasing the toxicity of MWCNTs on nematodes by increasing the expression of *mir-259* (Fig. 10).

In conclusion, in this study, we investigated the molecular basis for *mir-259* in regulating MWCNTs toxicity. In nematodes, activation of the *mir-259* mediated a protection mechanism for animals against the MWCNTs toxicity on longevity and aging related phenotypes. During the control of MWCNTs toxicity, RSKS-1 acted as an important target for *mir-259. mir-259* acted in the pharynx-intestinal valve and *rsks-1* acted in the pharynx to regulate MWCNTs toxicity. Moreover, RSKS-1 functioned upstream of the AAK-2-DAF-16 signaling cascade and suppressed the function of this signaling cascade to regulate MWCNTs toxicity. The *C. elegans mir-259* is the homologue of human *miR-216*^34^. Our results highlight the important role of miRNAs mediated protection mechanisms for animals against the adverse effects from environmental toxicants.

## Methods

### Characterization of MWCNTs

MWCNTs (diameter: 10–20 nm, length: 6–15 μm) were from Shenzhen Nanotech. Port Co. Ltd (Shenzhen, China). MWCNTs morphology in K-medium (50 mM NaCl, 30 mM KCl, 10 mM NaOAc, pH 6.0) was examined by transmission electron microscopy (TEM, JEM-200CX, JEOL, Japan) (Fig. S2a). Length distribution of MWCNTs was shown in Fig. S2b. Fourier transform infrared spectroscopy (FTIR) of MWCNTs was determined (Avatar 370, Thermo Nicolet, USA). The peak at 3367 cm^−1^ in the spectra of MWCNTs was attributed to -OH groups, the peak at 2850 cm^−1^ in the spectra of MWCNTs was attributed to C-H (sp3), the peak at 1629 cm^−1^ in the spectra of MWCNTs was attributed to C-H stretching, the peak at 1053 cm^−1^ in the spectra of MWCNTs was attributed to C-O stretching, and the peak at 2916 cm^−1^ in the spectra of MWCNTs was attributed to the stretching vibrations of alkyl groups (Fig. S2c). The FTIR spectra of MWCNTs implies the normal chemical structure on the surface of MWCNTs after sonication. Zeta potential of MWCNTs was analyzed by Nano Zetasizer (Nano ZS90, Malvern Instrument, UK). Zeta potential of MWCNTs in K-medium was −33.4 ± 2.5 mV.

### *C. elegans* strains and exposure

Nematode strains used in this study contain wild-type N2, mutants of *mir-259*(*n4106)*, *rsks-1*(*ok1255)*, *aak-2*(*ok524)*, *daf-16*(*mu86), aak-2*(*ok524);rsks-1*(*ok1255)*, *daf-16*(*mu86);aak-2*(*om524*), and *rsks-1*(*ok1255*)*;mir-259*(*n4106*), and transgenic strains of *maIs268*[*mir-259::GFP*], *mir-259*(*n4106*)*Ex*(P*myo-2-mir-259*), *mir-259*(*n4106*)*Ex*(P*ges-1-mir-259*), *mir-259*(*n4106*)*Ex*(P*pie-1-mir-259*), *mir-259*(*n4106*)*Ex*(P*ref-*1*-mir-259*), *rsks-1*(*ok1255*)*Ex*(P*myo-2-rsks-1*), and *rsks-1*(*ok1255*)*Ex*(P*dpy-7-rsks-1*)*. mir-259*(*n4106*) is a loss-of-function mutation with the deletion. The deletion breakpoints are: GATTATAATGCAAACAACCTGGGGGATC/CAGTATCTTCA…AAGAGCGAAAGT/ACAGTCTCCTCCTTCTTTGCTCACTTCT. *rsks-1*(*ok1255*) is a loss-of-function mutation with the deletion of 1700 bp. *aak-2*(*ok524*) is a loss-of-function mutation with the deletion of 409 bp. *daf-16*(*mu86*) is a loss-of-function mutation with an 11 kb *daf-16* genomic deletion that removes nearly all of the winged-helix domain and ~1 kb upstream of the 5′ UTR. Some strains were purchased from *Caenorhabditis* Genetics Center (funded by NIH Office of Research Infrastructure Programs (P40 OD010440)). Gravid nematodes were maintained on nematode growth medium (NGM) plates seeded with *Escherichia coli* OP50, and were lysed with a bleaching mixture (0.45 M NaOH, 2% HOCl) after washing animals off the plates into centrifuge tubes^13^. Synchronous L1-larvae nematodes were prepared as described^35^.

### Exposure and toxicity assessment

MWCNTs were dispersed in K medium to prepare a stock solution (1 mg/mL). The stock MWCNTs solution was sonicated for 30 min (40 kHz, 100 W), and diluted to the used concentration (1 mg/L) with K medium just prior to exposure. Prolonged exposure to MWCNTs was performed from L1-larvae to young adults in 12-well sterile tissue culture plates at 20 °C in the presence of food (OP50). After MWCNTs exposure, the nematodes were used for the toxicity assessment using endpoints of lifespan and locomotion behavior.

Lifespan was assayed at 20 °C basically as described^36^,^37^. During the lifespan assay, the hermaphrodite nematodes were transferred daily for the first 7 days of adulthood. Nematodes would be checked every two-day, and were scored as dead if they did not move even after repeated taps with a pick. Sixty nematodes were examined per treatment, and three replicates were performed.

Endpoints of head thrash and body bend were used to reflect the locomotion behavior of nematodes as described^38^,^39^. Head thrash and body bend were assessed under the dissecting microscope by eyes. A head thrash is defined as a change in the direction of bending at the mid body. A body bend is defined as a change in the direction of the part of the nematodes corresponding to the posterior bulb of the pharynx along the *y* axis, assuming that nematode was traveling along the *x* axis. Adult day-1 was used as a control for assay of locomotion behavior during aging. Twenty nematodes were examined per treatment, and six replicates were performed.

### Bioinformatics analysis for targeted gene prediction of *mir-259*

The corresponding targeted genes for *mir-259* were predicted using TargetScan version 6.2 (http://www.targetscan.org/worm_52/). TargetScan is a tool for searching for the presence of conserved sites that match seed region of a miRNA so as to predict the biological targets of certain miRNA.

### Reverse-transcription and quantitative real-time polymerase chain reaction (qRT-PCR)

Total RNAs extracted using RNeasy Mini kit (Qiagen) were reverse transcribed using PrimeScript™ RT reagent kit (Takara, Otsu, Shiga, Japan). Purity and concentration of RNAs were evaluated by OD260/280 in a spectrophotometer. cDNA synthesis was performed in a 12.5 μL reaction volume containing 625 ng total RNA, 0.5 mM reverse-transcript primers, 50 mM Tris-HCl, 75 mM KCl, 3 mM MgCl_2_, 10 mM dithiothreitol, 20 units ribonuclease inhibitor, and 100 U reverse transcriptase (Takara, China). After cDNA synthesis, real-time PCR was performed using SYBR Premix Ex Taq™ (Takara) for the amplification of the examined gene. Real-time PCR was performed using primers for target gene of *rsks-1* (forward primer, 5′-CCGTTTGTGGGATTCACC-3′; reverse primer, 5′-TGGCTTTCTCGGGCTCTT-3′), and reference gene of *tba-1* (forward primer, 5′-TCAACACTGCCATCGCCGCC-3′; reverse primer, 5′-TCCAAGCGAGACCAGGCTTCAG-3′). Relative quantification of targeted gene of *rsks-1* in comparison to reference *tba-1* gene was determined, and the final results were expressed as relative expression ratio between targeted gene and reference gene. All reactions were performed in triplicate.

### DNA constructs and germline transformation

To generate entry vector carrying promoter sequence, promoter region for *ges-1* gene specially expressed in the intestine, *myo-2* gene specially expressed in the pharynx, *pie-1* gene expressed in the reproductive tract, *ref*-1 gene specially expressed in the valve between pharynx and intestine, or *dpy-7* gene specially expressed in the hypodermis was amplified by PCR from wild-type *C. elegans* genomic DNA. These promoter fragments were inserted into pPD95_77 vector in the sense orientation. *rsks-1/Y47D3A.16* cDNA or *mir-259* was amplified by PCR, and inserted into corresponding entry vector carrying the *ges-1*, *myo-2*, *pie-1*, *ref*-1, or *dpy-7* promoter sequence. Germline transformation was performed by coinjecting testing DNA at the concentration of 10–40 μg/mL and marker DNA of P*lin-44::gfp* at the concentration of 60 μg/mL into the gonad of nematodes as described^40^.

### Distribution and translocation of MWCNTs

To investigate the distribution and translocation of MWCNTs, Rho B was loaded on MWCNTs by mixing Rho B (1 mg/mL, 0.3 mL) with MWCNTs (0.1 mg/mL, 5 mL) as described previously^20^. The unbound Rho B was removed by dialysis against the distilled water over 72-h. The prepared MWCNTs/Rho B was stored at 4 °C before use. Nematodes were incubated with MWCNTs/Rho B at the concentration of 1 mg/L for 3-h. After incubation, nematodes were washed with M9 buffer for three times. The exposed nematodes were analyzed under a fluorescence microscopy. Rho B treatment was used as the control. Twenty nematodes were examined per treatment.

### Statistical analysis

Data were expressed as means ± standard deviation (SD). Graphs were prepared with Microsoft Excel software (Microsoft Corp., Redmond, WA). Statistical analysis was performed using SPSS 12.0 software (SPSS Inc., Chicago, USA), and the differences between groups were determined using analysis of variance (ANOVA). The probability levels of 0.05 and 0.01 were considered to be statistically significant. The lifespan data were analyzed using a 2-tailed 2 sample t-test (Minitab Ltd, Coventry, UK).

## Additional Information

**How to cite this article**: Zhuang, Z. *et al.* Function of RSKS-1-AAK-2-DAF-16 signaling cascade in enhancing toxicity of multi-walled carbon nanotubes can be suppressed by *mir-259* activation in *Caenorhabditis elegans. Sci. Rep.* **6**, 32409; doi: 10.1038/srep32409 (2016).

## Supplementary Material

Supplementary Information
