# Supplementary material for: Function of RSKS-1-AAK-2-DAF-16 signaling cascade in enhancing toxicity of multi-walled carbon nanotubes can be suppressed by mir-259 activation in Caenorhabditis elegans
Source: Sci Rep. 2016 Aug 30;6:32409. doi: 10.1038/srep32409 (PMC5004105; doi:10.1038/srep32409)
Supplement: Supplementary Information [file srep32409-s1.doc]

**Function of RSKS-1-AAK-2-DAF-16 signaling cascade in enhancing toxicity of multi-walled carbon nanotubes can be suppressed by *mir-259* activation in *Caenorhabditis elegans***

Ziheng Zhuang^a, b, *^, Min Li^a, c^, Hui Liu^a^, Libo Luo^b^, Weidong Gu,^b^ Qiuli Wu^c^ & Dayong Wang^c, *^

^a^School of Pharmaceutical Engineering and Life Sciences, Changzhou University, Changzhou 213164, China.

^b^Changzhou No. 7 People’s Hospital, Changzhou 213011, China

^c^Key Laboratory of Environmental Medicine Engineering in Ministry of Education, Medical School, Southeast University, Nanjing 210009, China

^*^Corresponding author. E-mail address: cczuzzh@163.com (Z. Zhuang), [dayongw@seu.edu.cn](mailto:dayongw@seu.edu.cn) (D. Wang).

**Supporting Information:**


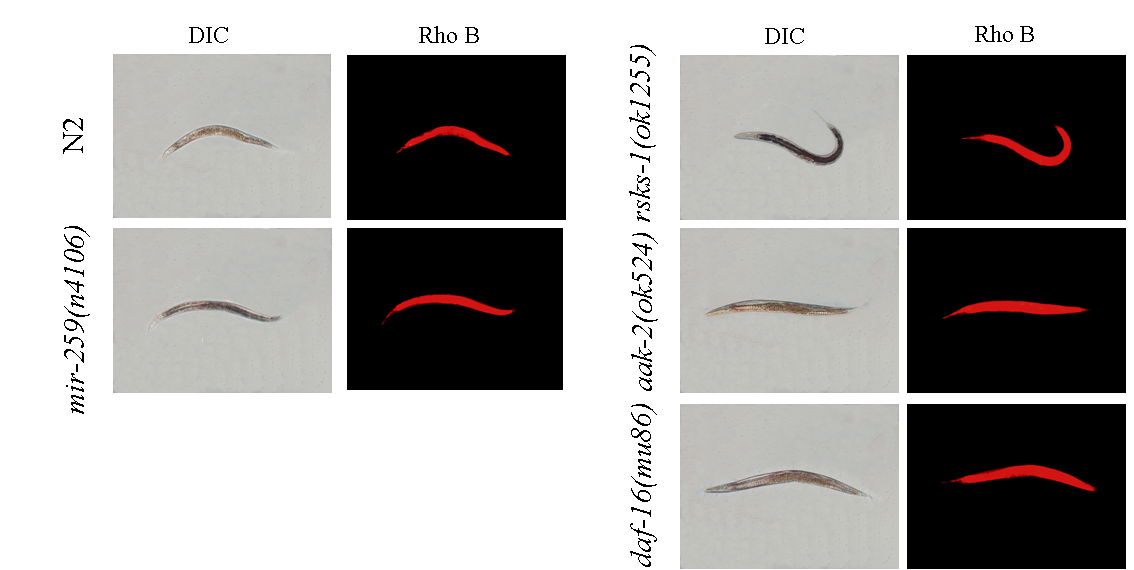


**Figure S1. Distribution and translocation of Rho B in nematodes.**


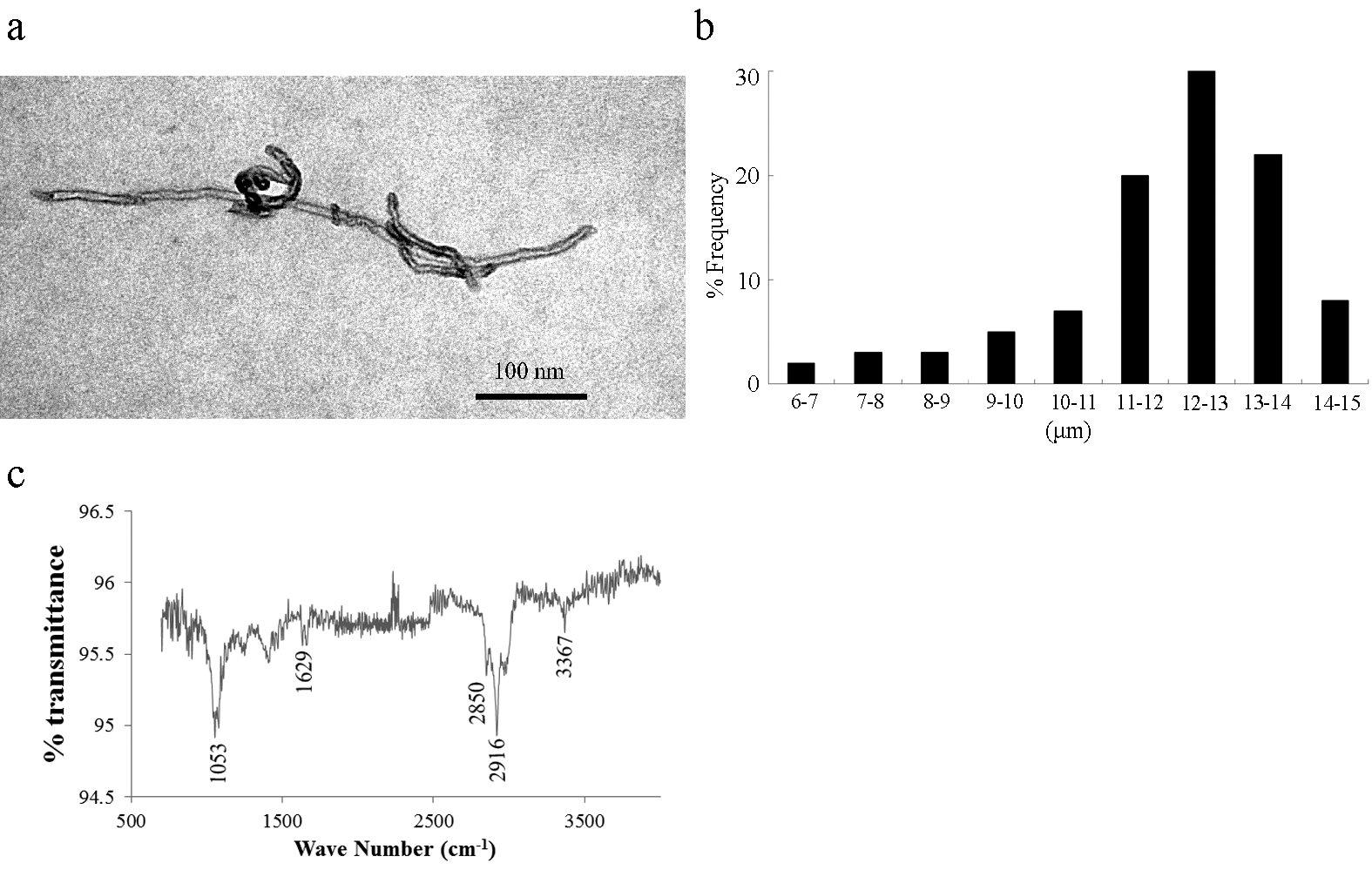


**Figure S2. Properties of MWCNTs.** (**a**) TEM picture of MWCNTs in K-medium after sonication. (**b**) Length distribution of MWCNTs after sonication. (**c**) FTIR spectra of MWCNTs after sonication.
